# Human primary airway epithelial cells isolated from active smokers have epigenetically impaired antiviral responses

**DOI:** 10.1186/s12931-016-0428-2

**Published:** 2016-09-07

**Authors:** Wenxin Wu, Wei Zhang, J. Leland Booth, David C. Hutchings, Xiaoqiu Wang, Vicky L. White, Houssein Youness, Cory D. Cross, Ming-Hui Zou, Dennis Burian, Jordan P. Metcalf

**Affiliations:** 1 Pulmonary and Critical Care Division, Department of Medicine, University of Oklahoma Health Sciences Center, Oklahoma City, OK USA; 2 Cherokee Healthcare Services, Catoosa, OK USA; 3 Center of Molecular and Translational Medicine, Georgia State University, Atlanta, GA USA; 4 Civil Aerospace Medical Institute, Federal Aviation Administration, Oklahoma City, OK USA; 5 Department of Microbiology and Immunology, University of Oklahoma Health Sciences Center, Oklahoma City, OK USA; 6 Veterans Affairs Medical Center, Oklahoma City, OK USA

**Keywords:** RIG-I, TLR3, Interferon, Influenza, HBEC, COPD, Human, Lung, Innate immunity

## Abstract

**Background:**

Cigarette smoking (CS) is the main risk factor for the development of chronic obstructive pulmonary disease (COPD) and most COPD exacerbations are caused by respiratory infections including influenza. Influenza infections are more severe in smokers. The mechanism of the increased risk and severity of infections in smokers is likely multifactorial, but certainly includes changes in immunologic host defenses.

**Methods:**

We investigated retinoic acid-inducible protein I (RIG-I) and interferon (IFN) induction by influenza A virus (IAV) in human bronchial epithelial cells (HBEC) isolated from smokers or nonsmokers. Subcultured HBEC cells were infected with A/Puerto Rico/8/1934 (PR8) IAV at an MOI of 1. After 24 h of infection, cells and supernatants were collected for qRT-PCR, immunoblot or ELISA to determine RIG-I, Toll-like receptor3 (TLR3) and IFN expression levels.

**Results:**

IAV exposure induced a vigorous IFN-β, IFN-λ 1 and IFN-λ 2/3 antiviral response in HBEC from nonsmokers and significant induction of RIG-I and TLR3. In cells from smokers, viral RIG-I and TLR3 mRNA induction was reduced 87 and 79 % compared to the response from nonsmokers. CS exposure history was associated with inhibition of viral induction of the IFN-β, IFN-λ1 and IFN-λ 2/3 mRNA response by 85, 96 and 95 %, respectively, from that seen in HBEC from nonsmokers. The demethylating agent 5-Aza-2-deoxycytidine reversed the immunosuppressive effects of CS exposure in HBEC since viral induction of all three IFNs was restored. IFN-β induction of RIG-I and TLR3 was also suppressed in the cells from smokers.

**Conclusion:**

Our results suggest that active smoking reduces expression of antiviral cytokines in primary HBEC cells. This effect likely occurs via downregulation of RIG-I and TLR3 due to smoke-induced epigenetic modifications. Reduction in lung epithelial cell RIG-I and TLR3 responses may be a major mechanism contributing to the increased risk and severity of viral respiratory infections in smokers and to viral-mediated acute exacerbations of COPD.

## Background

Chronic obstructive pulmonary disease (COPD) is a major worldwide cause of morbidity and mortality. According to the World Health Organization, by 2030, COPD will be the third most common global cause of death [1]. Cigarette smoking (CS) is the main risk factor for the development of COPD, which is a chronic inflammatory disease characterized by progressive, partially reversible airflow limitation [2]. CS alters innate and adaptive immune responses, and it has been proposed that many of the deleterious health consequences of CS are due to its adverse immune effects [3]. Respiratory viral infections with influenza virus, rhinoviruses, and respiratory syncytial virus are leading pathogens associated with COPD exacerbations, which are associated with progressive loss of lung function [4]. In fact, 64 % of COPD exacerbations are caused by respiratory infections including influenza and respiratory syncytial virus [5]. Additionally, many studies have established a relationship between CS and the risk of influenza infection [6, 7]. The mechanism of the increased risk and severity of infections in smokers is probably multifactorial, but certainly includes changes in alteration of immunologic host defenses.

The innate immune system responds to influenza A virus (IAV) through three classes of microbial pathogen sensors, called pattern recognition receptors (PRRs). Most cells use the cytosolic sensor, retinoic acid inducible gene I (RIG-I), to detect IAV and trigger antiviral responses [8]. Endosomal based Toll-like receptors (TLRs) are also involved in the recognition of, and response to IAV. TLR3, a double-strand RNA sensor, may be used by some epithelial cells to detect the viral replicative intermediate dsRNA [9]. Plasmacytoid dendritic cells (pDCs) use TLR7 to recognize influenza genomic RNA, upon its release in late endosomes [10]. Finally, the PRR nucleotide-binding domain and leucine-rich-repeat-containing proteins (NLRP), including NLRP3 and nucleotide-binding oligomerization domain 2 (NOD2), play multiple roles in regulating the innate immune response during virus infection through modulation of inflammasome activation, antiviral activity, and priming of adaptive immunity [11–14].

Airway epithelial cells are the primary location of replication, and also represent the first line of defense against IAV by eliciting an innate immune response [15, 16]. In airway epithelial cells, RIG-I is the central regulator of IAV-mediated induction of antiviral cytokines [17]. TLR3 also contributes in the response to IAV in epithelial cells [18]. We have demonstrated that RIG-I and TLR3 are the two major PRRs induced by IAV infection in A549 and human primary type II alveolar epithelial cells (AEC) [19]. Either of them may compensate to maintain antiviral immunity when the other signaling mechanism is shut down. One of the major downstream products of RIG-I and TLR3 signaling is the interferon (IFN) cytokine family. IFNs are further divided into type I (mainly IFN-α and β), II (IFN-γ) and III (IFN-λ) subtypes, based in part on the differential use of unique receptors through which they mediate signal transduction to induce antiviral activity. Rapid production of type I and III IFN is a central and essential component of the antiviral response in airway epithelial cells [20, 21]. We have previously shown that RIG-I is crucial for the induction of the early antiviral cytokine response, and cigarette smoke extract (CSE) inhibits the RIG-I initiated innate immune response to IAV [22–24].

Although galectin-3 and the receptor for advanced glycation end products (RAGE) are involved in the innate response to IAV [25–27], there is no evidence that these PRRs recognize IAV and initiate cytokine responses to this virus in human epithelial cells. An extensive immunohistochemical survey of normal human tissues showed RAGE expression in type II alveolar pneumocytes, endothelia and alveolar macrophages [28]. TLR7 and galection-3 mainly recognize pathogens in dendritic cells [29]. In this study, we will focus on the two major PRRs, RIG-I and TLR3, that recognize IAV in epithelial cells and compare antiviral cytokine induction by IAV in primary human bronchial epithelial cells (HBEC) from smokers and nonsmokers.

## Methods

### Isolation of primary human bronchial epithelial cells

Human bronchial epithelial cells (HBEC) were obtained by bronchoscopy and bronchial brushing with the written, informed consent from both smoking and non-smoking, healthy, adult volunteers in accordance with a protocol approved by the Institutional Review Board of the University of Oklahoma Health Sciences Center (IRB # 2197). The smokers had a smoking history of at least 10 pack years with ½ to 1 pack of cigarettes per day. All smoking and nonsmoking participants were gender, age and ethnicity matched. Three or four separate bronchi were brushed and the cells were rinsed from the brush into 10 ml sterile saline until 5 × 10^6^ to 1 × 10^7^ cells total were collected as determined by hemocytometer counts for total and viable cells by trypan blue exclusion. The HBECs were centrifuged at 400 × g for 5 min. Cells were resuspended to 5 × 10^5^ cells/ml in complete Bronchial Epithelial Cell Growth Medium (BEGM; Lonza Group Ltd.); were seeded into collagen coated tissue culture plates (Bio-Coat, BD Biosciences) at a density of 1×10^5^ cells/cm^2^ and were propagated in an incubator at 37 °C in 5 % CO_2_. After 24 h the cells were washed with HBSS to remove non-adherent cells and fresh complete BEGM was added. When the cultures were near confluence (7–10 days), the monolayers were lifted with 1x Accutase solution and were subcultured at a 1:5 dilution. After each passage the cells grew to confluence within 4 to 5 days, and when the cultures were split, freezer stocks were prepared in 80 % BEGM + 10 % fetal bovine serum + 10 % DMSO and were stored in liquid nitrogen vapor at −190 °C. Cells were rapidly thawed, washed in BEGM, and cultured till confluence prior to experiments.

### Preparation of influenza virus stock

Influenza virus, A/PR/34/8 (PR8), was passaged in Madin–Darby canine kidney (MDCK) cells. Virus was grown in MDCK cells in DMEM/F12 with ITS+ (BD Biosciences, Franklin Lakes, NJ) and trypsin, harvested at 72 h postinfection and titered by plaque assay in MDCK cells. There was no detectable endotoxin in the final viral preparations used in the experiments as determined by limulus amebocyte lysate assay (Cambrex, Walkersville, MD). The lower limit of detection of this assay is 0.1EU/ml or approximately 20 pg/ml LPS.

### Preparation of CSE

One (100 mm) cigarette without filter was combusted with a pump. The smoke was bubbled through 25 ml of cell medium at a speed of 50 ml/min. The resulting suspension was filtered through a 0.22-μm pore filter (Lida Manufacturing, Kenosha, WI) to remove bacteria and large particles. This solution, considered to be 100 % CSE, was diluted and applied to cell cultures within 30 minutes of preparation. The nicotine concentration of 100 % CSE was 73.48 ± 1.08 μg/ml.

### Measurement of mRNA expression by quantitative real-time PCR (qRT-PCR)

Total RNA from cells was extracted using a modified TRIzol (Invitrogen, Carlsbad, CA) protocol, spectrophometrically quantitated, and the integrity verified by formaldehyde agarose gel electrophoresis. Equal amounts (1 μg) of RNA from each sample were used with oligo (dT) as primers for production of cDNA (SuperScript II First-Strand Synthesis System for RT-PCR, Invitrogen, Carlsbad, CA). Gene specific primers for the PRRs, cytokines and the β-actin housekeeping genes were used. qRT-PCR was performed using 100 ng sample RNA and SYBR Green (Quanta Biosciences, Gaithersburg, MD) in a Bio-Rad CFX96™ Touch Real-Time PCR Detection System. Results were calculated and graphed from the comparative C_T_ method (ΔΔCT C_T_ method). The primers’ sequences were as follows: RIG-I forward 5′- TCCTTTATGAGTATGTGGGCA -3′; RIG-I reverse 5′- TCGGGCACAGAATATCTTTG -3′; IFN-β forward 5′- GCTCTCCTGTTGTGCTTCTCCAC -3′; IFN-β reverse 5′- CAATAGTCTCATTCCAGCCAGTGC -3′; β-actin forward 5′- GCCAACCGCGAGAAGATGACC-3′; β-actin reverse 5′- CTCCTTAATGTCACGCACGATTTC-3′; TLR3 forward 5′-GTCTGGGAACATTTCTCTTC-3′; TLR3 reverse 5′-GATTTAAACATTCCTCTTCGC-3′; IFN-λ1 forward 5′- CGCCTTGGAAGAGTCACTCA-3′; IFN-λ1 reverse 5′- GAAGCCTCAGGTCCCAATTC-3′; IFN-λ2/3 forward 5′- AGTTCCGGGCCTGTATCCAG-3′; IFN-λ2/3 reverse 5′- GAGCCGGTACAGCCAATGGT-3′; IP-10 forward 5′-TCTAGAACCGTACGCTGTACCTGC-3′; IP-10 reverse 5′-CTGGTTTTAAGGAGATCT-3′; IRF7 forward 5′-CAGATCCAGTCCCAACCAAG-3′; IRF7 reverse 5′- GTCTCTACTGCCCACCCGTA-3′.

### ELISA and multiplex immunoassay

ELISAs of IP-10 and IFN-λ2/3 cytokine protein levels in the supernatants were all performed using commercially available kits (R & D system, Minneapolis, MN).

### siRNA transfection of HBEC

For siRNA treatment, cells were plated 24 h before treating with siRNAs (Ambion). siRNAs were diluted in 250 μl of Opti-MEM medium and mixed gently. Five μl of Lipofectamine 2000 (Invitrogen) was added in 250 μl Opti-MEM medium and incubated for 5 min. Diluted siRNA and Lipofectamine 2000 were combined and mixed gently and incubated for 20 min at RT. The siRNA-Lipofectamine 2000 complexes were added to each well and mixed gently. siRNA final concentration was 20 nM. The cells were then incubated at 37 °C for 48 h prior to use in experiments.

### RIG-I protein determination by immunoblotting

The cells were harvested and homogenized, and then lysed in 500 μl of cold lysis buffer (150 mM NaCl, 50 mM Tris, pH 8.0, 10 mM EDTA, NaF, sodium pyrophosphate, 1 % NP-40, 0.5 % sodium deoxycholate, 0.1 % SDS, 10 μg of leupeptin/ml). Cell homogenates were clarified by centrifugation at 10,000 × g, at 4 °C for 10 min, and the clarified lysates were mixed with SDS-PAGE sample buffer (60 mM Tris, pH 6.8, 10 % glycerol, 2.3 % SDS) and heated to 95 °C for 5 min. The samples were separated by 4–15 % gradient gel and electrophoretically transferred to polyvinylidene fluoride (PVDF) membranes. For the detection of proteins, the membranes were immunoblotted with rabbit polyclonal antibody specific for RIG-I (Abcam, Cambridge, MA) and GAPDH (R & D Systems). The membranes were then treated with horseradish peroxidase-labeled goat anti-rabbit IgG (Cell Signaling Technology, Beverly, MA) and chemiluminescent reagents (Pierce Biotechnology, Rockford, IL). Blots were developed using the Syngene G:box Bioimaging System and GeneTools software (Syngene, Frederick, MD) and resultant signals were quantified using ImageQuant software (BD/Molecular Dynamics, Bedford, MA).

### Statistical analysis

Where applicable, the data have been expressed as the means ± standard error of the mean (SEM). Statistical significance was determined by one-way ANOVA with Student-Newman-Keuls *post hoc* correction for multiple comparisons. Significance was considered as *P* < 0.05.

## Results

### HBEC isolated from smokers have suppressed antiviral responses during influenza infection

**Fig. 1:**
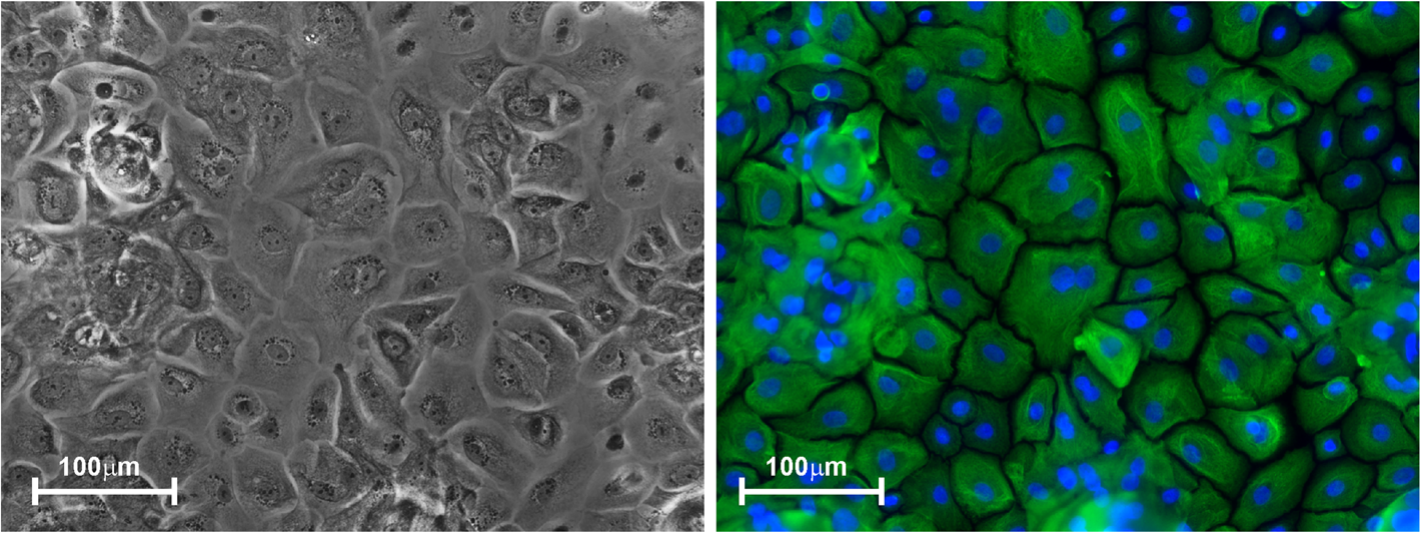
Isolation and culture of primary HBEC. HBEC are shown in a transmitted light image (left panel) and immune-stained for pan-cytokeratin (right panel, green = cytokeratin, blue = nuclear DNA). The antibodies used were anti-human pan-cytokeratin rabbit polyclonal (Life Technologies, DP010-05) and anti-rabbit IgG –Cy3 conjugate (Jackson Immuno Research Laboratories, 711-166-152). Bar = 100 μm

We isolated and purified primary HBEC from participant volunteers with normal lung function, and no recent history of viral illness. The cells have the cobblestone morphology (Fig. 1a) and positive immunostaining for pan-cytokeratin (Fig. 1b), typical of epithelial cells. In subsequent experiments described below, HBEC were subcultured for approximately five days before viral infection in order to study the innate cytokine response to IAV PR8.

**Fig. 2:**
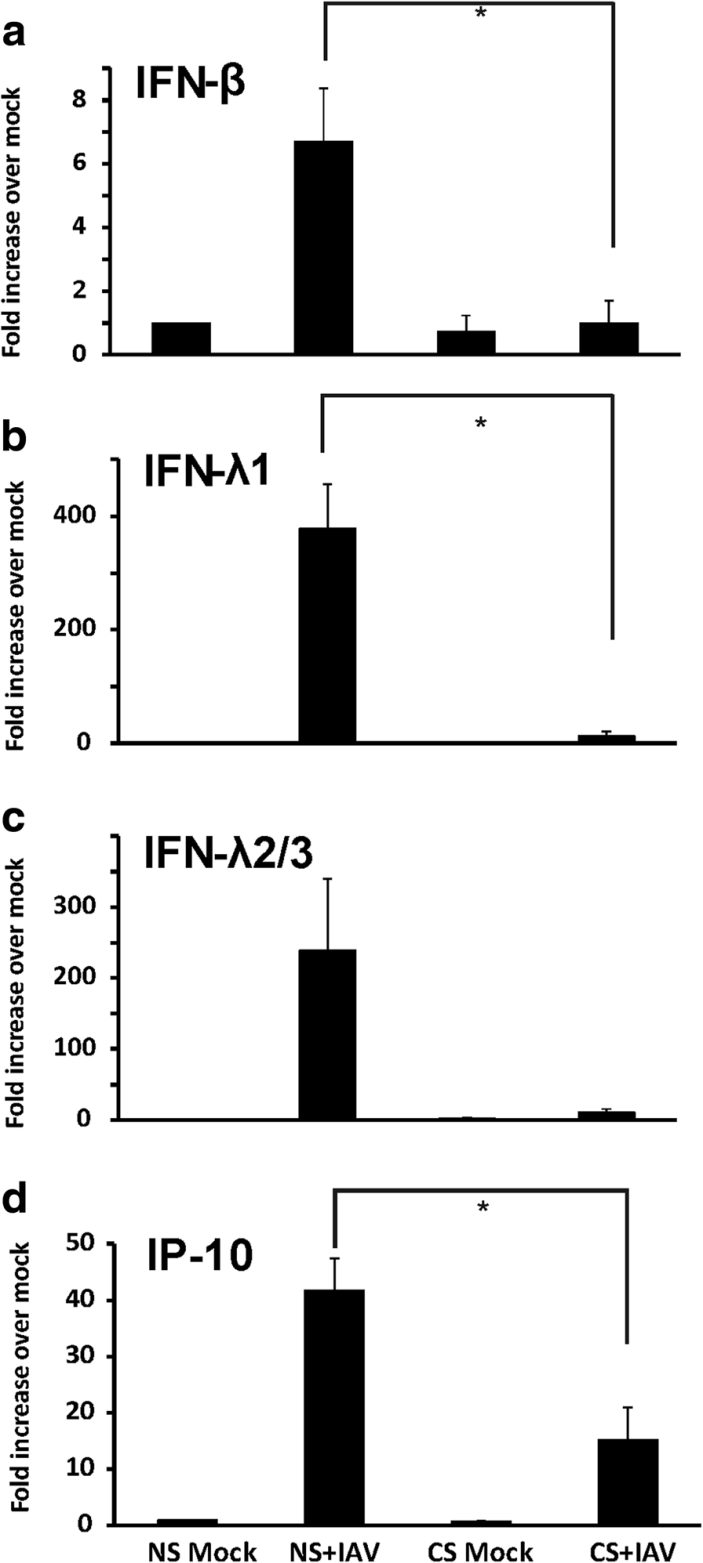
Antiviral interferons and IP-10 induced by influenza virus infection were suppressed in HBEC isolated from smokers. After infection of cells with IAV PR8 at the MOI of 1 for 24 h, total RNA was extracted and cell cytokine mRNA expression was assessed by qRT-PCR. Transcript levels of the cytokines (**a**) IFN-β, (**b**) IFN-λ1, (**c**) IFN-λ2/3, and (**d**) IP-10 were normalized relative to the constitutively expressed β-actin gene. CS = cigarette smoker; NS = nonsmoker. Data were expressed as the means ± SEM from three separate experiments from different donors. * denotes significant difference between the indicated groups, *P* < 0.05

**Fig. 3:**
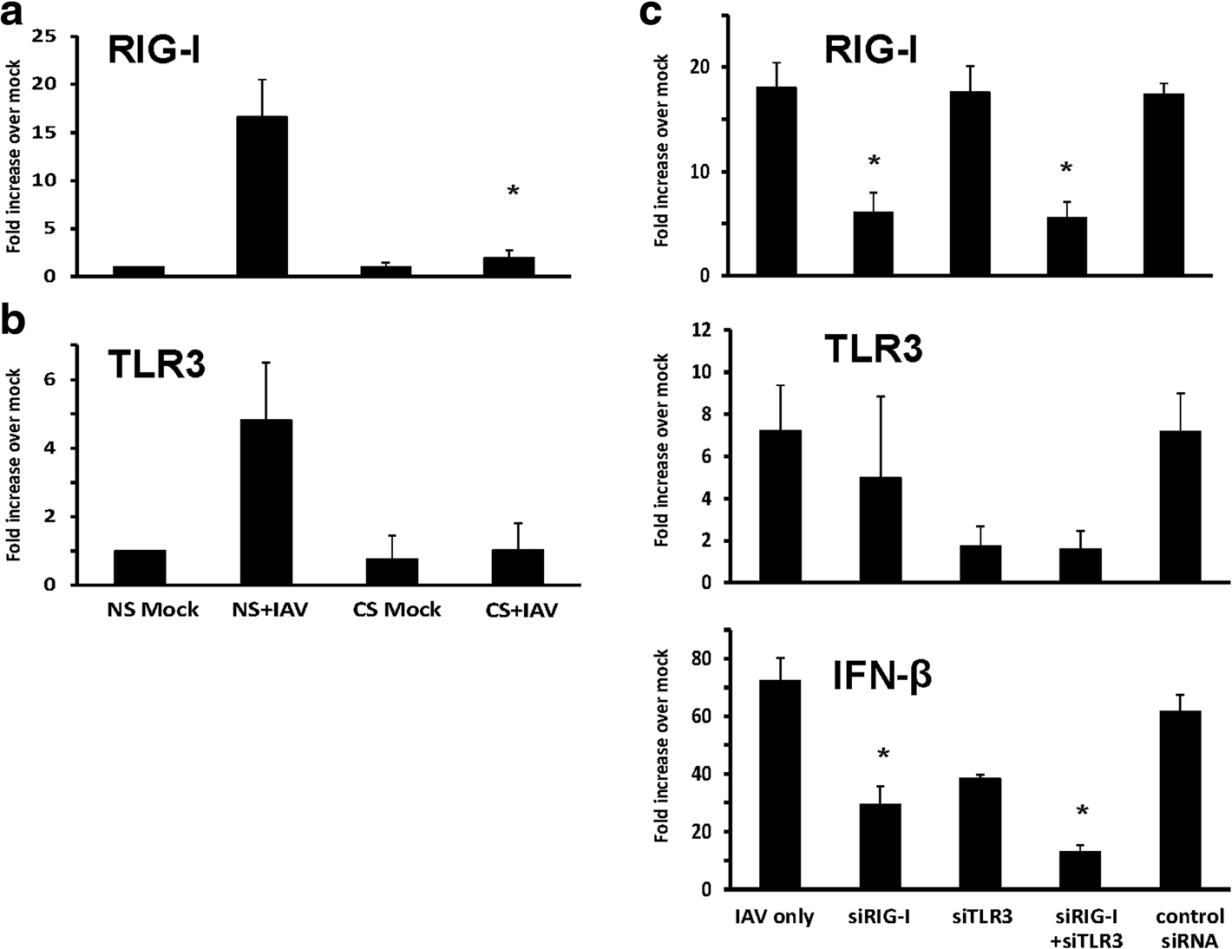
RIG-I and TLR3 upregulation by influenza virus infection was required for IFNβ induction and was suppressed in HBEC isolated from smokers. After infection of cells with IAV at an MOI of 1 for 24 h, total RNA was extracted and cell PRR mRNA expression was assessed by qRT-PCR. Transcript levels of RIG-I (**a**) and TLR3 (**b**) were normalized relative to the constitutively expressed β-actin gene. **﻿(c) ** HBEC from nonsmokers were first transfected with RIG-I or TLR3 siRNA for two days before IAV infection. Statistical significance was determined by ANOVA. Data were expressed as the means ± SEM from three separate experiments from different donors. * denotes significant difference compared to data from the IAV infected cells from nonsmokers (NS + IAV), *P* < 0.05

First, we compared the HBEC antiviral response to IAV from current smokers and healthy nonsmokers. HBEC isolated from smokers and nonsmokers were exposed to 6 × 10^6^ PFU/ml of IAV for 24 h. Virus-free diluents (mock) were the negative controls for the experiments. As anticipated, IAV exposure induced a vigorous antiviral cytokine response in HBEC from nonsmokers. Expression of the antiviral cytokines IFN-β, IFN-λ1 and IFN-λ2/3 mRNA were increased 7 fold, 380 fold and 240 fold, respectively, over mock infection. However, in cells from smokers, smoking inhibited viral induction of the IFN-β, IFN-λ1 and IFN-λ 2/3 mRNA response by 85, 96 and 95 % (Fig. 2a-c), respectively, from that seen in HBEC from nonsmokers. IP-10 is an important antiviral chemokine due to its ability to recruit activated monocytes, T lymphocytes and natural killer cells to the sites of infection. The IP-10 mRNA antiviral response was also suppressed in HBEC from smokers by 63 % (Fig. 2d). In human lung epithelial cells, RIG-I and TLR3 are the two most important PRRs for triggering antiviral immune responses to IAV. We then examined the effect of prior CS exposure on RIG-I and TLR3 mRNA expression in these cells (Fig. 3). Both RIG-I and TLR3 had decreased IAV stimulated mRNA expression in smokers although the reduction was not statistically significant for TLR3. After 24 h of infection, IAV induced RIG-I expression was decreased by 87 % while TLR3 was decreased 79 % in cells from smokers. In prior work, we have shown signaling through both RIG-I and TLR3 is important for IFN induction by IAV in human lung epithelial cells [19]. To determine whether RIG-I and TLR3 signaling is important in IFN responses in HBEC, we knocked down these PRRs in HBEC using siRNAs and assessed IFN responses to IAV (Fig. 3c). First, we confirmed siRNA inhibition of RIG-I and TLR3. RIG-I and TLR3 mRNA induction by IAV was blocked 67 and 78 %, respectively, in the corresponding siRNA treated cells. IFN-β mRNA induction was decreased 60 and 48 % in RIG-I or TLR3 siRNA treated, IAV infected cells compared to control siRNA treated cells. Double knockdown of RIG-I and TLR3 almost completely blocked IFN-β induction by IAV (82 % reduction). These results showed that RIG-I and TLR3 are critical to IFN induction by IAV in HBEC and that CS exposure inhibited RIG-I and TLR3 induction by IAV and caused immunosuppression of antiviral cytokine responses.

**Fig. 4:**
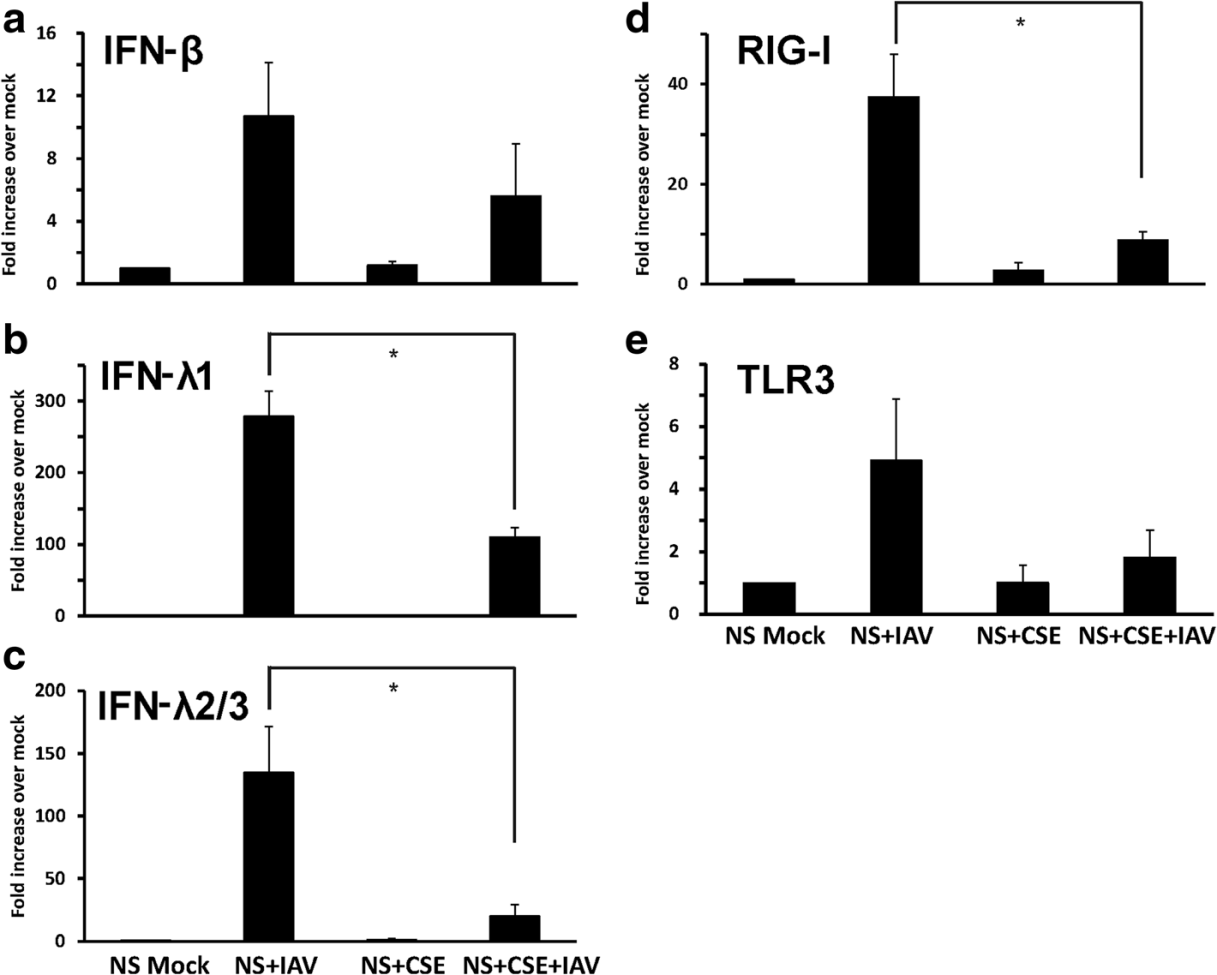
Antiviral cytokine and pattern recognition receptor induction by influenza virus infection was inhibited in CSE-treated HBEC isolated from nonsmokers. HBEC were cultured in growth media with 2 % CSE for 24 h. After infection of the cells with IAV at an MOI of 1 for 24 h, total RNA was extracted, and cell cytokine, (**﻿a**-**c**﻿), ﻿and PRR, (**﻿d**, **e﻿**), mRNA expression was assessed by qRT-PCR. Transcript levels were normalized relative to the constitutively expressed β-actin gene. Data were expressed as the means ± SEM from three separate experiments from different donors. * denotes significant difference between the indicated groups, *P* < 0.05

To mimic the CS effect on HBEC ex vivo, we also treated HBEC isolated from nonsmokers with 2 % CSE, using the same methods described earlier [23]. Cultured cells were treated with 2 % CSE or not for 24 h prior to infection with 6 × 10^6^ PFU/ml of IAV. Virus-free diluents (mock) were the negative controls for the experiments. Consistent with our previous findings, pretreatment of the cells with 2 % CSE decreased IAV stimulated IFN-β, IFN-λ1 and IFN-λ 2/3 mRNA response by 48, 61 and 85 % compared to cells without CSE treatment (Fig. 4a-c). The effect of CSE on the HBEC RIG-I and TLR3 response to IAV was also examined (Fig. 4d, e). CSE reduced RIG-I and TLR3 mRNA induction by 76 and 63 %, respectively. Thus, the experiments demonstrated that CSE suppresses antiviral cytokine responses in IAV-infected HBEC through inhibition of RIG-I and TLR3 induction.

**Fig. 5:**
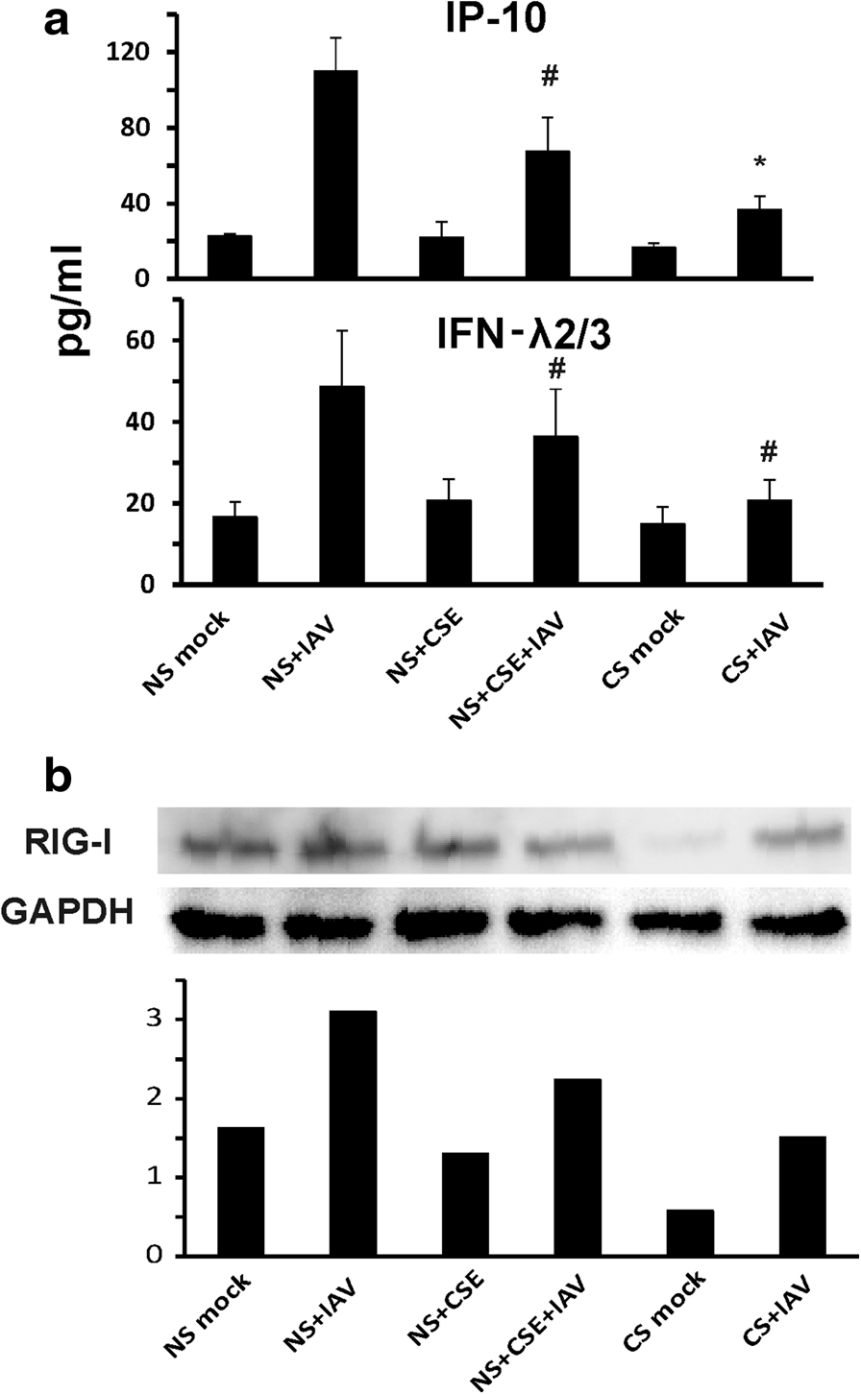
HBEC isolated from smokers have impaired IAV-stimulated antiviral cytokine and RIG-I protein induction. The cells from nonsmokers were treated with or without 2 % CSE for 24 h. After infection of HBEC cells from nonsmokers and smokers with IAV at an MOI of 1 for 24 h, supernatants and cells were collected. (**a**) Cytokine IP-10 and IFN λ2/3 expression in the supernatant was assessed by ELISA. (**b**) RIG-I protein expression in HBEC was detected by immunoblotting, normalized to GAPDH. Data were expressed as the means ± SEM from four separate experiments from different donors. * denotes significant difference compared to data from the IAV infected cells from nonsmokers (NS + IAV), *P* < 0.05. # denotes no significant difference compared to data from the IAV infected cells from nonsmokers (NS + IAV)

To confirm the qRT-PCR result at the level of protein expression, we determined IP-10 and IFN-λ 2/3 protein levels in cultured cell supernatants by ELISA. Unstimulated HBEC supernatants contained 22 pg/ml IP-10 and 16 pg/ml IFN-λ2/3 as basal levels. IAV infection increased these levels to 105 and 51 pg/ml respectively (Fig. 5a). Cell pretreatment with 2 % CSE reduced IAV stimulated IP-10 and IFN-λ2/3 levels to 67 pg/ml and 39 pg/ml, respectively (Fig. 5a, middle two bars). More importantly, cells from smokers had significantly lower IAV induced IP-10, at 37 pg/ml (*p* < 0.05) and much less IFN-λ 2/3, at 17 pg/ml (no significant difference) as compared with IAV stimulated, non-CSE exposed HBEC from nonsmokers (Fig. 5a, right two bars labeled CS are cells from smokers). We also confirmed that in vivo CS exposure inhibited the viral RIG-I protein response (Fig. 5b, CS + IAV vs. NS + IAV). Pretreatment with 2 % CSE of cells from nonsmokers showed a comparable suppressive effect as CS on IAV-mediated RIG-I protein induction (Fig. 5b, NS + CSE + IAV vs. NS + IAV). These results demonstrate CSE exposure inhibited RIG-I protein induction by influenza in cells similar to that seen in smokers, consistent with our results that CS might worsen viral infection through immunosuppression of the antiviral response.

**Fig. 6:**
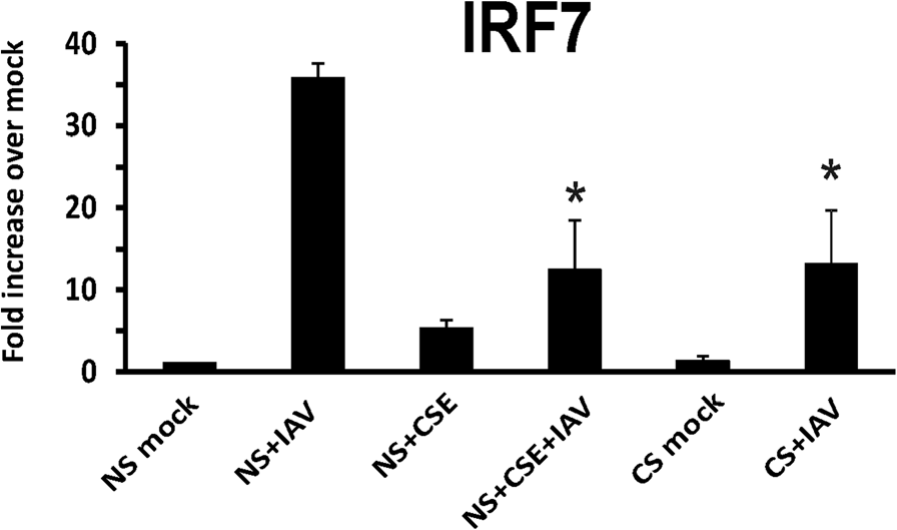
CS suppressed IRF7 mRNA induction by influenza virus in HBEC from smokers. HBEC from nonsmokers were cultured in growth media with or without 2 % CSE for 24 h. After infection of the cells with IAV at an MOI of 1 for 24 h, total RNA was extracted and IRF7 mRNA expression was assessed by qRT-PCR. Transcription level was normalized relative to the constitutively expressed β-actin gene. Data were expressed as the means ± SEM from three separate experiments from different donors. * denotes significant difference compared to data from the IAV infected cells from nonsmokers (NS + IAV), *P* < 0.05

Transcription factor IRF7-dependent amplification of type I and III IFNs is required for protection against primary infection by IAV in humans [30]. One research group demonstrated that CS resulted in reduced influenza-mediated induction of IRF7 in upper respiratory tract nasal epithelial cells (NECs) [31]. We next analyzed the IAV induced IRF7 expression in our lower respiratory tract cells from smokers and nonsmokers. Influenza induction of IRF7 mRNA expression was significantly reduced by 64 % in smokers comparing to nonsmokers (Fig. 6). CSE treatment, as compared with no CSE treatment, suppressed IAV-stimulated IRF7 mRNA induction by 65 % in HBEC from nonsmokers.

### Demethylation reversed the immunosuppressive effects of CS exposure on HBEC

**Fig. 7:**
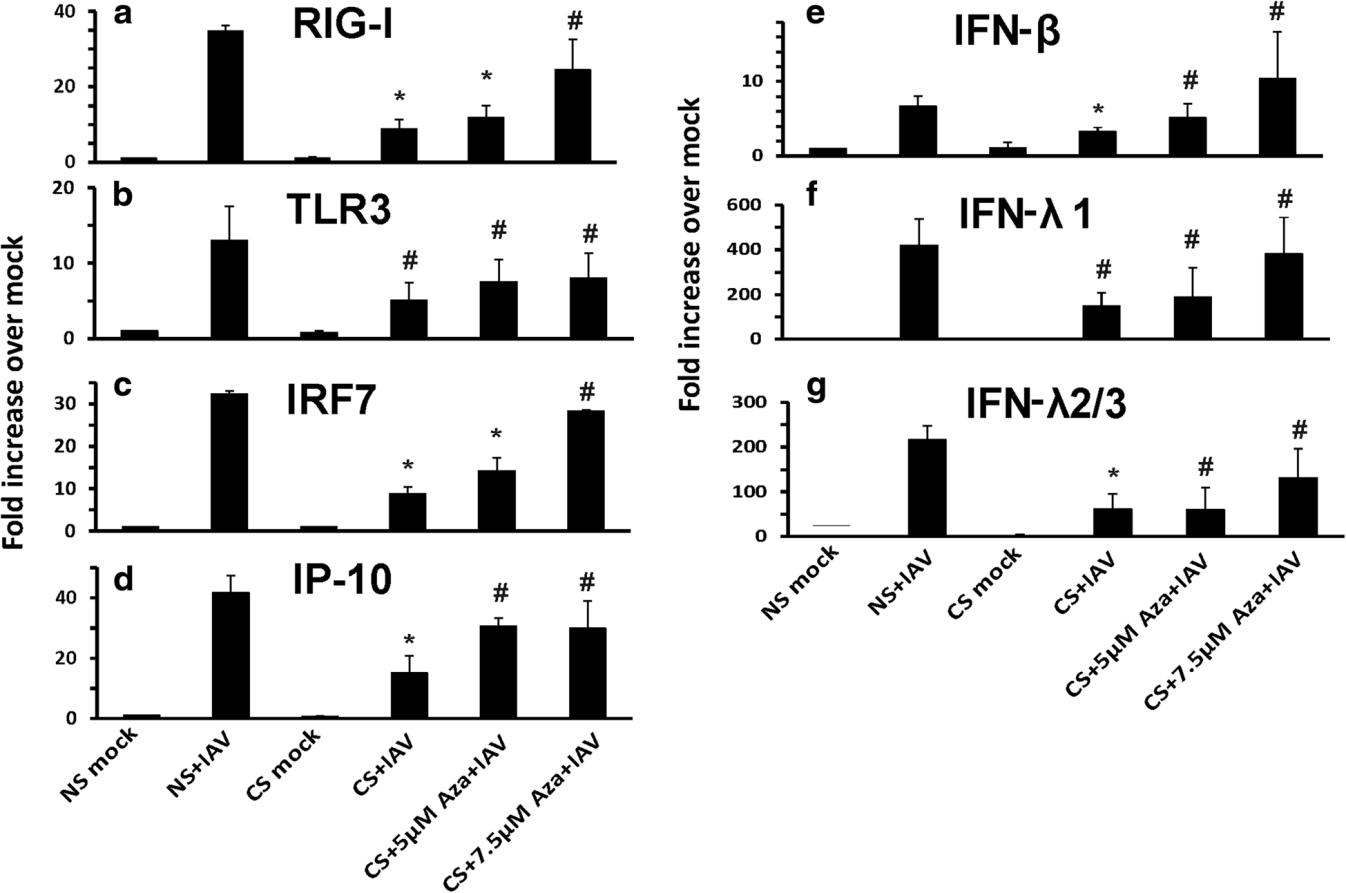
Demethylation restored the antiviral response to influenza virus in HBEC isolated from smokers. HBEC were treated with fresh 5 μM and 7.5 μM 5-Aza-2-deoxycytidine (5-Aza) each day for 3 days before IAV infection. After infection of the cells with IAV at an MOI of 1 for 24 h, total RNA was extracted and mRNA expression was assessed by qRT-PCR. Transcript levels of RIG-I (**a**), TLR3 (**b**), IRF7 (**c**), IP-10 (**d**), IFN-β (**e**), IFN-λ1 (**f**) and IFN-λ2/3 (**g**) were normalized relative to the constitutively expressed β-actin gene. Data were expressed as the means ± SEM from three separate experiments from different donors. * denotes significant difference compared to data from the IAV infected cells from nonsmokers (NS + IAV), *P* < 0.05. # denotes no significant difference compared to data from the IAV infected cells from nonsmokers (NS + IAV)

**Fig. 8:**
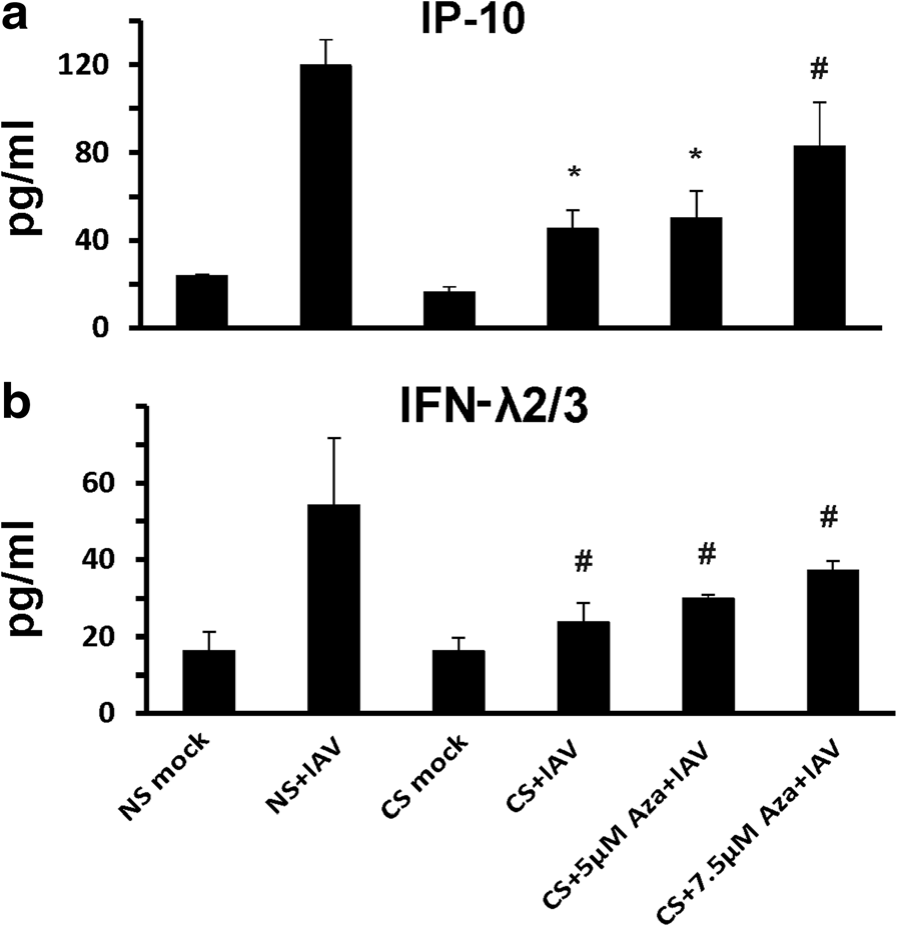
Demethylation restored IAV induced cytokine IP-10 (**a**) and IFN λ2/3 (**b**) protein expression in supernatants of HBEC isolated from smokers. HBEC were treated with fresh 5 μM and 7.5 μM 5-Aza each day for 3 days before IAV infection. After infection of the cells with IAV at an MOI of 1 for 24 h, supernatants were collected and cytokine expression was assessed by ELISA. Data were expressed as the means ± SEM from three separate experiments from different donors. * denotes significant difference compared to data from the IAV treated cells from nonsmokers, *P* < 0.05. # denotes no significant difference compared to data from the IAV infected cells from nonsmokers (NS + IAV)

Since the isolated HBEC from smokers have been passaged for several generations and still maintained the altered phenotypic response to IAV, we investigated whether CS may have induced epigenetic changes responsible for immunosuppression in these cells. We therefore performed experiments to ask if demethylation reverses the inhibition of the IAV response seen in cells from smokers. HBEC cells were preincubated with increasing amounts of the demethylating agent 5-Aza-2-deoxycytidine (5-Aza, Sigma) for 3 days before IAV infection. 5-Aza was added freshly each day and maintained throughout the experiment. IFN induction was measured at 24 h after IAV infection. As shown in Fig. 7, exogenous 5-Aza restored the RIG-I, TLR3, IRF7, IP-10 and IFN virus response in cells from smokers in a dose-dependent manner. After IAV infection, there was no significant difference in RIG-I, IP-10 and IFN induction between cells from nonsmokers and cells from smokers treated with 7.5 μM of 5-Aza (Fig. 7). The restoration of IP-10 and IFN-λ 2/3 responses to virus was also assessed at the protein level by ELISA (Fig. 8). Consistently, supernatant protein levels of the two cytokines reflected mRNA expression changes. Thus, CS suppressed expression of RIG-I and TLR3 and resultant suppression of antiviral cytokine responses is likely due to hypermethylated silenced genes in HBEC from smokers.

### Amplification of RIG-I and TLR3 induction by interferon was suppressed in HBEC from smokers

**Fig. 9:**
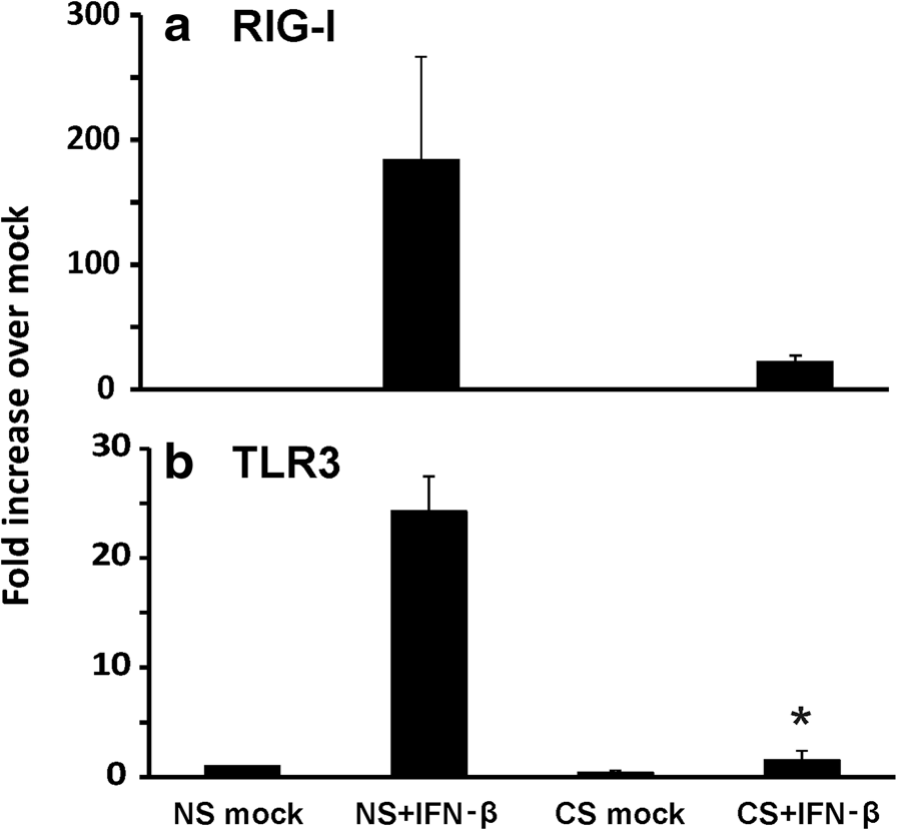
CS suppressed RIG-I and TLR3 transcriptional induction by IFN-β in HBEC from smokers. After HBEC cells were treated with 500U/ml of IFN-β for 6 h, total RNA was extracted and mRNA expression was assessed by qRT-PCR. Transcript levels of RIG-I (**a**) and TLR3 (**b**) were normalized relative to the constitutively expressed β-actin gene. Data were expressed as the means ± SEM from three separate experiments from different donors. * denotes significant difference compared to data from the IAV infected cells from nonsmokers (NS + IAV), *P* < 0.05

The RIG-I pathway is induced by RNA virus infection. Type I IFNs also stimulate antiviral responses by binding to a shared receptor (IFNAR). Besides IFN-stimulated gene induction by IFNα/β, the type I IFNs transcriptionally stimulate a second phase of RIG-I and TLR3 production. This autocrine/paracrine feedback amplification results in type I IFN activation of an antiviral state in nearby cells. We next sought to examine if RIG-I and TLR3 mRNA induction by IFN-β was affected by smoking. Cultured HBEC were treated with 500U/ml of IFN-β (PBL Assay Science) without virus infection for 6 h. The cells then were collected and mRNA induction was determined by quantitative RT-PCR. As expected, both RIG-I and TLR3 mRNA were significantly induced by IFN-β in cells from nonsmokers (Fig. 9). However, induction was suppressed in cells from smokers.

## Discussion

RIG-I and TLR3-initiated antiviral responses in infected human lung epithelial cells are well known for their ability to reduce viral replication, and both type I and type III IFNs inhibit influenza replication. This suppression was enhanced when both types of IFNs were simultaneously used [32]. We have previously demonstrated that RIG-I and TLR3 are the two major PRRs induced by IAV infection in human A549 and type II AEC [19]. In this report, we demonstrated that influenza-induced antiviral cytokine responses were epigenetically inhibited in primary HBEC from smokers compared with nonsmokers.

CS affects the first lines of defense by increasing airway epithelial permeability, causing tissue disruption, and decreasing mucociliary clearance [33]. Many studies have shown that CS exerts detrimental effects on immunologic host defenses by inhibition of stimulated cytokine production that may interfere with effective and efficient antimicrobial responses [34–36]. Eddleston et al. demonstrated that pretreatment of BEAS-2B cells or normal human bronchial epithelial cells with CSE diminished IP-10 and RANTES mRNA induction by either the viral mimic polyinosine-polycytidylic acid (Poly I:C) or human rhinovirus 16 [37]. Another group showed that CSE-mediated inhibition of poly I:C-induced antiviral innate responses in human peripheral blood mononuclear cells (PBMC) is mainly due to inhibition of IFN-β production [38]. Here, we directly compared the innate response from primary HBEC obtained from smokers with these responses from HBEC from nonsmokers. We demonstrated that HBEC from smokers have a diminished RIG-I and TLR3-initiated antiviral response to influenza infection compared to nonsmokers. It has been demonstrated that active smoking reduces both human lung macrophage expression of TLR3, and dsRNA-induced IP-10 production [39]. However, the expression of mRNA transcripts for nucleic acid receptors by RT-PCR was only measured in resting macrophages without viral stimulation. Our data showed diminished RIG-I and TLR3 mRNA expression in HBEC with IAV stimulation, which is more applicable to actual IAV infection in vivo as these cells are a major site of viral replication. Our research provides further insights into mechanisms by which CS alters epithelial innate immune responses to virus infection. These observations could help to explain the increased susceptibility of cigarette-smoking humans to severe respiratory viral infections.

Our results suggested CS also inhibits autocrine/paracrine amplification of RIG-I and TLR3 induction by IFNs (Fig. 9) in surrounding cells. This may occur through epigenetic modification of the promoters of RIG-I and TLR3 by cigarette smoke, inhibiting binding of activating transcription factors. Epigenetic modifications, mainly DNA methylation and histone modification, regulate gene expression by changing DNA accessibility and chromatin structure without changing the DNA coding sequence. DNA methylation, the attachment of methyl groups to DNA, potentially alters gene expression profiles of CS-exposed target cells [40, 41]. Earlier work from Jaspers et al. found that methylation of IRF7, which is a transcriptional factor in the IFN response, was associated with an impaired antiviral defense response in human NECs from smokers [31]. Our data supports this previous study, and provides further mechanistic information. Using HBEC cells from smokers, our study demonstrated that the demethylation agent 5-Aza reversed the suppressed RIG-I, TLR3, IRF7 and IFN response to IAV. RIG-I and TLR3 play important roles in the recognition of, and response to, IAV in human lung epithelial cells. Since IRF7 is downstream of RIG-I-initiated signaling, epigenetic modifications of RIG-I might provide another mechanism for IRF7 inhibition found by Jaspers. Our work is consisted with previous preliminary data demonstrating that HBEC from subjects with COPD have minimal RIG-I induction and impaired IFN responses to IAV [42]. In our report, we examined both RIG-I and TLR3 expression and revealed that smoke-induced epigenetic changes are responsible for this effect. The immunosuppressive effects of CS exposure are likely due to increased methylation (i.e., hypermethylation) of the promoters of RIG-I and TLR3. It is known that CS alters the methylation pattern of the genome. Recently, many studies have investigated epigenetic changes relating to exposure to cigarette smoke that lead to lung cancer [43–45]. Few studies have, however, reported on the epigenetic changes in the human immune system that may lead to compromised antiviral activity with resultant enhanced viral replication and prolonged recovery. One study found that lung tumors of patients with COPD differ from those of patients without COPD, with differentially methylated and expressed genes being mainly involved in the immune response [46]. Our future studies will focus on whether there are DNA methylation changes in human lung epithelial cells from smokers that alter host defense responses.

## Conclusion

Taken together, we have shown CS affects the inductive and amplification phases of the RIG-I and TLR3 mediated antiviral response to IAV in lung airway epithelial cells. We chose IAV PR8 for these studies, as this mouse adapted virus stain is the standard H1N1 strain used in both human and animal studies. However, it is recognized that it is not an unmodified fresh clinical isolate, which might limit the applicability of our conclusions relative to other IAV. Further work needs to be done to study the epigenetic impact of CS exposure and its effect on gene expression of important human innate immune components.
